# Bioartificial Therapy of Sepsis: Changes of Norepinephrine-Dosage in Patients and Influence on Dynamic and Cell Based Liver Tests during Extracorporeal Treatments

**DOI:** 10.1155/2016/7056492

**Published:** 2016-06-28

**Authors:** Martin Sauer, Jens Altrichter, Cristof Haubner, Annette Pertschy, Thomas Wild, Fanny Doß, Thomas Mencke, Maren Thomsen, Johannes Ehler, Jörg Henschel, Sandra Doß, Stephanie Koch, Georg Richter, Gabriele Nöldge-Schomburg, Steffen R. Mitzner

**Affiliations:** ^1^Departments of Anesthesiology and Intensive Care Medicine, Medical Faculty of the University of Rostock, 18057 Rostock, Germany; ^2^Departments of Medicine, Division of Nephrology, Medical Faculty of the University of Rostock, 18057 Rostock, Germany; ^3^Departments of Surgery, Medical Faculty of the University of Rostock, 18057 Rostock, Germany; ^4^Fraunhofer Institute for Cell Therapy and Immunology, 04103 Leipzig, Germany

## Abstract

*Purpose.* Granulocyte transfusions have been used to treat immune cell dysfunction in sepsis. A granulocyte bioreactor for the extracorporeal treatment of sepsis was tested in a prospective clinical study focusing on the dosage of norepinephrine in patients and influence on dynamic and cell based liver tests during extracorporeal therapies.* Methods and Patients.* Ten patients with severe sepsis were treated twice within 72 h with the system containing granulocytes from healthy donors. Survival, physiologic parameters, extended hemodynamic measurement, and the indocyanine green plasma disappearance rate (PDR) were monitored. Plasma of patients before and after extracorporeal treatments were tested with a cell based biosensor for analysis of hepatotoxicity.* Results.* The observed mortality rate was 50% during stay in hospital. During the treatments, the norepinephrine-dosage could be significantly reduced while mean arterial pressure was stable. In the cell based analysis of hepatotoxicity, the viability and function of sensor-cells increased significantly during extracorporeal treatment in all patients and the PDR-values increased significantly between day 1 and day 7 only in survivors.* Conclusion.* The extracorporeal treatment with donor granulocytes showed promising effects on dosage of norepinephrine in patients, liver cell function, and viability in a cell based biosensor. Further studies with this approach are encouraged.

## 1. Introduction

Severe sepsis and septic shock remain a major cause of morbidity and mortality in critically ill patients and the treatment of these patients is very expensive [1–4]. The impairment of hemodynamics and liver function are major problems in patients with severe sepsis [5, 6]. In patients with septic shock a liver dysfunction or liver failure occurred in nearly 19% and lead to a poor prognosis of these patients [6, 7].

Extracorporeal therapies have been suggested to influence successfully immune imbalances and subsequently the clinical course of multiorgan failure and sepsis [8]. Some studies showed hemodynamic stabilization of patients during extracorporeal treatment of sepsis; however, no clear impact on survival was seen [8, 9]. The influence of extracorporeal therapies of sepsis on liver function has not been investigated yet, but it may be an important tool for the improvement of outcome in this high-risk cohort population with liver dysfunction or liver failure [6].

Extracorporeal bioreactors were studied in the treatment of liver failure and acute renal failure associated with sepsis using hepatocytes or renal tubular cells; the proper choice of the cell-source was of central importance [10–14]. The use of immune cells (leukocytes) to treat sepsis in an extracorporeal setting was reported from our group [15]. With regard to cellular immunocompetence, functional impairment of neutrophils and monocytes is associated with increased mortality in advanced stages of sepsis [16–23]. Therefore, we developed an extracorporeal granulocyte bioreactor system [24, 25]. The rationale for such an approach is that on one hand the plasma-modifying capacity of human phagocytes can be used (e.g., to remove antigenic material from the circulation) while on the other hand control over these cells can be maintained (e.g., retention of the cells and their release and break-down products, preventing local tissue effects; 15). In in vitro studies and two large animal models for septic shock, we were able to show the proof of principle and promising survival data [15, 24, 26]. Additionally, the granulocyte bioreactor was studied in a pilot phase I trial with ten septic shock patients and showed safety and compatibility of this complex therapy [25]. During extracorporeal granulocyte treatments, the dosage of norepinephrine could be significantly reduced, as blood pressure was stable in the treated septic patients.

The focus of the current work was to document the exact impact of the extracorporeal granulocyte treatment on the dosage of norepinephrine in patients and the liver function using extended hemodynamic monitoring with the PiCCO-System and dynamic measurement of the liver function with the LiMON-System [27, 28]; moreover, in this second clinical study, cell based analysis of hepatotoxicity of plasma of patients [29, 30] with severe sepsis or septic shock patients was done. Based on the results of the second study, a first controlled study with this new therapy should be designed.

## 2. Materials and Patients

The study was conducted in accordance with the Helsinki Declaration, received ethics approval from the local research ethics committee (reg.-no: II HV 28/2003), and was notified to the state authorities according to German pharmaceutical and medical device law. The trial was registered https://clinicaltrials.gov/ (reg.-no: NCT00818597). Written informed consent was obtained from all participants or from the patients' representatives if direct consent could not be received.

### 2.1. Patients

Between January 2010 and November 2011, ten patients of one medical and one surgical intensive care units of a tertiary care university hospital were enrolled in the study. During a 22-month period, all patients were screened for the parameters of severe sepsis or septic shock as defined by international consensus criteria [31]. Definitions of organ dysfunctions were adopted from the PROWESS study [32] with the difference that liver failure was not an exclusion criterion in this current study. The exclusion criteria were age under 18 years, hepatitis C, HIV infection, and active bleeding or contraindications against systemic heparinization.

### 2.2. Procedures

**Figure 1:**
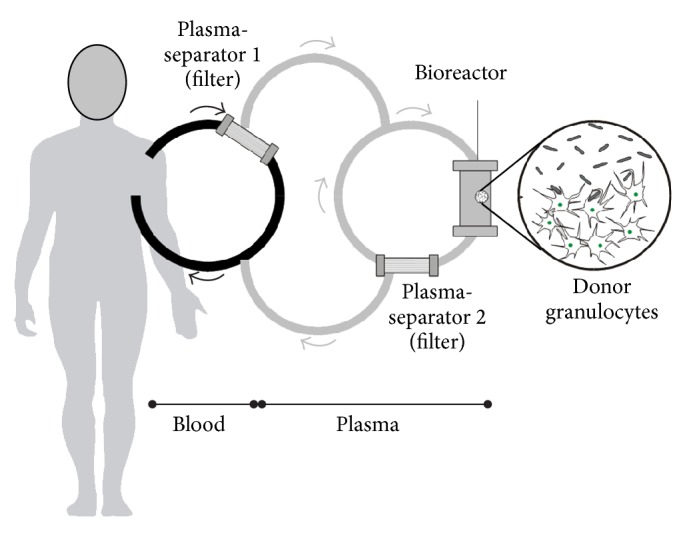
Schematic drawing of the extracorporeal plasma separation and cell perfusion model.

After inclusion of a patient, a healthy blood donor for obtaining an ABO-compatible granulocyte concentrate was identified and stimulated with corticosteroids (each 8 mg p.o. methylprednisolone, Sanofi-Aventis Deutschland GmbH, Frankfurt, Germany) and lenograstim (Granocyte, each 1.5 *μ*g/kg s.c., Chugai Pharma Deutschland, Frankfurt, Germany) 16 h before donation. Granulocytes were collected by extracorporeal density gradient centrifugation using hydroxyethyl starch (HES 200/0.5 6%, Fresenius Kabi AG, Bad Homburg, Germany) and citrate in a cell separator (COBE Spectra, Gambro BCT, Planegg-Martinsried, Germany) according to standard procedures. Prior to the treatment, the inclusion criteria were reconfirmed and the patients were treated for up to six hours with an extracorporeal bioreactor (Figure 1) consisting of plasma separation and plasma perfusion through the cell compartment containing the donor cells. Blood access was venovenous via a Shaldon catheter. Plasma separation was carried out by a dialysis monitor (BM25, Edwards Lifesciences GmbH, Unterschleissheim, Germany) using a 0.5 *μ*m pore-size plasma filter (PF 1000N, Gambro Hospal GmbH, Planegg-Martinsried, Germany). The plasma was infused into a continuously recirculating donor cell compartment that was prefilled with hemofiltration solution HF-BIC 35-410 (Fresenius Medical Care, Bad Homburg, Germany). Plasma reflux to the patient was done through a second PF 1000N plasma filter to withhold the donor cells from being infused into the patient. Total extracorporeal volume was 400 mL. The blood flow rate was 110–150 mL/minute with a plasma separation rate of 16.7–33.3 mL plasma/minute using the BM 25 monitor. The MARS-Monitor 1 TC (Gambro Rostock GmbH, Rostock, Germany) was used for the recirculating bioreactor circuit at a rate of 200 mL/minute and to maintain the temperature in the cell compartment at 37°C. Unfractionated heparin (40 IU/kg, Roche, Grenzach-Wyhlen, Germany) was given at the beginning of the extracorporeal treatment followed by a continuous infusion into the circuit. Heparin administration was adjusted to maintain activated clotting time (ACT) within 180–200 seconds. Following safety assessment of the first treatment, all patients were treated a second time 48 hours after the first treatment, again for up to six hours with granulocytes from the same donor.

### 2.3. Measurements

We recorded basic demographic information, illness severity (APACHE II, SOFA, MODS, and SAPS II scores), microbiological results, premorbidity, and clinical outcome for study cohort (Tables 1 and 2). Patients were followed up for 28 days and hospital survival. At the days “inclusion”, 1–8, 10, 12, 14, 21, 28, and before/after an extracorporeal bioreactor-treatment, the patients were screened for clinical and immunological data: hemodynamic, inflammation, coagulation, hemolysis, temperature, organ function blood parameters, cytokines, complement (C3, C4), and number of HLA-DR molecules per monocyte surface. “Day 1” was defined as the day of the first bioreactor-treatment. At inclusion, at days 1 to 7 and before and after extracorporeal treatment, hemodynamic monitoring was done with the PiCCO-System (Table 4, PULSION Medical Systems, Feldkirchen, Germany; 27). The LiMON-System (based on the indocyanine green plasma disappearance rate, PULSION; 28) was used for the dynamic measurement of the liver function before and after extracorporeal treatment and on day 7.

### 2.4. Cell Based Analysis of Hepatotoxicity: Cell Cultures and Test Methods

Before and after extracorporeal treatment, 10 mL plasma was drawn from each patient for testing with our hepatotoxicity test (biosensor; 29, 30). The method to determine the toxicity of patient plasma used the human hepatocytes cell line HepG2/C3A obtained from the American Type Culture Collection (ATCC CRL-10741). The cells were cultivated in Dulbecco's modified Eagle's Medium (GIBCO Life Technologies, Eggenstein, Germany). HepG2/C3A cells were seeded in 24-well cell culture plates in a density of 250.000 cells/well; then, the cells were cultured for three days with 1 mL heparinized plasma from subjects. Subsequently, cells were rinsed once with medium and incubated with fresh medium (1 mL) for three days. Cells, respectively, cell culture supernatants, were tested for viability (XTT-test: dehydrogenases activity in the mitochondria, cell-count and vitality with Trypan blue-staining), synthesis of microalbumin, and cytochrome 1A2 activity. Each test batch with plasma from test persons was duplicated (+medium control) and each measurement was taken twice. Microalbumin was determined nephelometrically from cell culture medium supernatant (Immage 800, Beckmann Coulter GmbH, Krefeld, Germany). The XTT-test was carried according to the protocol of Scudiero et al. [33]. The activity of cytochrome P450 1A2 was determined by means of O-demethylation of 7-ethoxyresorufine to resorufin. The measurement was carried according to the protocol of Kelly and Sussman [34].

### 2.5. Statistical Analysis

The Statistical Package for the Social Sciences (SPSS) was used to conduct nonparametric analyses using Friedman test and Wilcoxon test for the comparison of parameters in the course of disease; statistical significance between the survivors and nonsurvivors was analyzed with the Kruskal-Wallis one-way and the two-tailed Mann-Whitney* U* test. The results are expressed as the median with 0.25–0.75 quartile. Differences were considered significant at *p* < 0.05. Box Plots were used for graphics. The horizontal line within the boxes represents the median, whereas the upper part represents the 75th and the lower part the 25th percentiles. The whiskers represent the range of the values, whereas the circles and the asterisks show the outliers (extreme values that derivate from the rest of the sample).

## 3. Results

### 3.1. Clinical Characteristics of Patients and Survival

Nine patients with septic shock and one patient with severe sepsis were included in the study (all male, 9 out of 10 surgical patients). Details concerning diagnoses, source of infection, organ failure, age, sex, premorbidity, and survival are shown in Table 1. Nine out of ten patients had positive microbial tests; 18 Gram-positive bacteria, 12 Gram-negative bacteria, and 4 positive cultures with fungi were found.

The observed mortality rate was 40% within 28 days and 50% during stay in hospital. Five patients could be discharged from the hospital in stable condition. Patients 2 and 4 died after reduction of therapy on palliative care (on the very same day). During the first extracorporeal treatment, patient 6 died; an autopsy revealed an advanced and longer existing ischemia of the bowel. The time between beginning of shock and beginning of extracorporeal treatment was 5.5 (4.3–7.5) days. In Table 2, age, scores, and results of laboratory parameters, of hemodynamic monitoring (PiCCO-System), and of dynamic measurement of liver function (LiMON-System) at inclusion or at day 1 of survivors and nonsurvivors are shown. The dosage of norepinephrine, the values of bilirubin, and the median SAPS II were significantly higher and the indocyanine green plasma disappearance rate (ICG/PDR)/ICG-PDR cardiac index ratio was significantly lower in nonsurvivors than in survivors. Three patients developed a liver failure in the course of disease (Table 1, all nonsurvivors).

### 3.2. Extracorporeal Treatment

All extracorporeal treatments were carried out for 6 h without technical problems. Eight patients were treated twice within 72 h with an extracorporeal bioreactor containing 12.3 (10.4–14.4) x10E10 granulocytes from healthy donors. On average, 11.7 (10.3–12.0) liters of separated plasma were treated by the therapeutic donor cells. To test whether the donor cells were still functional, every two hours cells from the cell circuit were evaluated for viability and functionality. For the whole treatment, the cells showed a viability of more than 90% and unimpaired cellular functions like phagocytosis and oxidative burst (data not shown).

There was no significant change in coagulations markers (platelet counts, antithrombin, prothrombin time, and fibrinogen) within 12 h after the extracorporeal circulation. D-dimers did not increase significantly during the extracorporeal treatment (data not shown). No hemorrhages and no signs of hemolysis were observed. Haptoglobin remained within the normal range and no significant change in values of lactatedehydrogenase and potassium was seen during the treatments (data not shown). Moreover, no allergic reactions were recognized.

### 3.3. Inflammation

During the six-hour treatment, a dramatic increase in the number of leukocytes was observed (before: 14.9 (13.8–19.4); after 6 hours: 18.2 (14.6–24.8); x10E9/l; *p* = 0.002); after 12 hours, however, a decrease to baseline was seen. This increase during extracorporeal treatment was not due to changes in a particular subset of WBC, the ratio of segmented to banded neutrophils remained unchanged. During the extracorporeal treatments, the complement factors C3 and C4 decreased slightly but were even in normal ranges during the whole observation time (data not shown). The values of procalcitonin decreased significantly during the six-hour treatment (before: 6.9 (0.3–11.8); after 6 hours: 6.2 (0.3–9.4); ng/mL; *p* = 0.003) and between days 3 and 28 (all time points) compared with day 1 (before extracorporeal treatment, *p* < 0.05, data not shown). The expression per cell of HLA-DR on monocytes increased significantly from inclusion [6974 (5888–9119)] to day 4 [9278 (6394–12526)] and between inclusion and days 5 to 28 (all time points, *p* < 0.05, data not shown). Between day 1 (before extracorporeal treatment) and day 3 after extracorporeal treatment, the values of IL-6 and IL-8 increased; these changes, however, were not significant and the values normalized on day 8 (Table 3). The values of IL-10 and TNF-alpha were lower and decreased between day 1 and day 8 (Table 3).

### 3.4. Hemodynamic Parameters during Extracorporeal Treatment

**Figure 2:**
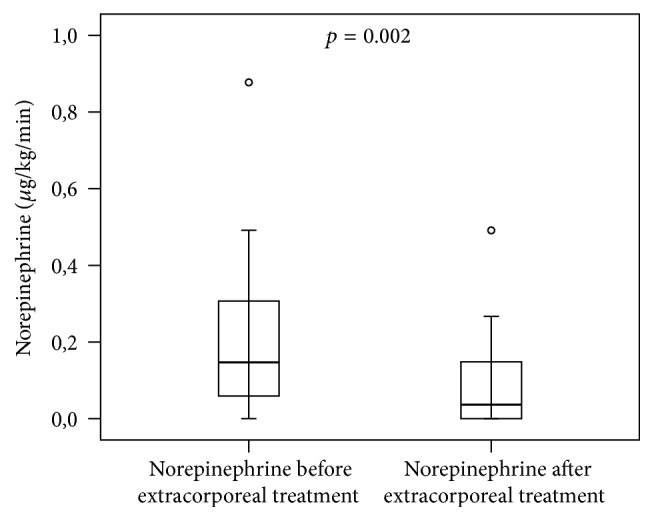
The dosage of norepinephrine (*μ*g/kg/min) could be reduced significantly during the extracorporeal granulocyte treatments (Mann-Whitney* U* test; *n* = 10; median/0.25–0.75 quartile).

The dosage of norepinephrine could be reduced significantly during the extracorporeal treatments (Figure 2). Additionally, during the extracorporeal treatments, the mean arterial pressure, the heart rate, the parameters of hemodynamic monitoring using the PiCCO-System, the central venous oxygen saturation, and values of lactate showed no significant changes; but the cardiac index and stroke volume index increased (Table 4). After the second extracorporeal treatments of patients, the use of norepinephrine could reduce continue in the course of the observation time (data not shown).

### 3.5. Liver Function Markers and Tests

The dynamic measurement of liver function used the LiMON-System (based on the indocyanine green plasma disappearance rate, ICG-PDR) and the ICG-PDR cardiac index ratio which both on day 1 showed significantly lower values in nonsurvivors than in survivors (Table 2). During the extracorporeal treatments, no significant increases of PDR-values and of ICG-PDR cardiac index ratio could be observed (Table 4). The PDR-values increased significantly (*p* = 0.027) between day 1 [11.9 (5.3–13.8)] and day 7 [23.3 (20.2–23.8)] only in surviving patients.

At inclusion, the values of bilirubin were significantly lower in survivors than in nonsurvivors (Table 2). In the course of disease, no significant differences of bilirubin-values were seen between the survivors and nonsurvivors and during extracorporeal treatments.

**Figure 3:**
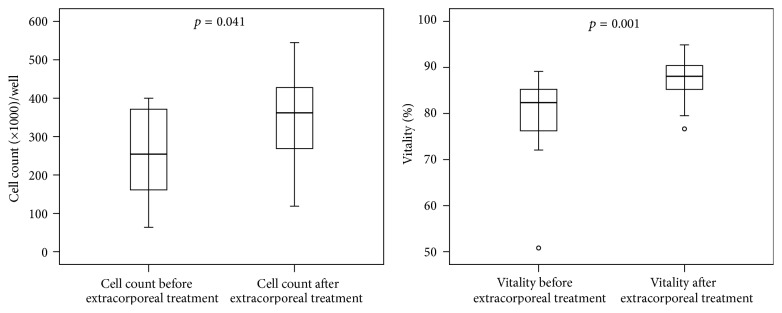
Cell-count and vitality (Trypan blue-staining) of HepG2/C3A cells (biosensor-test) incubated with plasma from septic patients before extracorporeal treatments compared to plasma from patients after extracorporeal granulocyte treatments (Mann-Whitney* U* test; *n* = 10; median/0.25–0.75 quartile).

**Figure 4:**
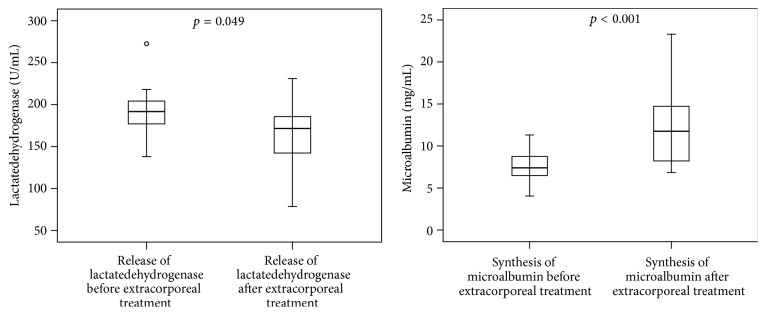
Release of lactatedehydrogenase and synthesis of microalbumin of HepG2/C3A cells (biosensor-test) incubated with plasma from septic patients before extracorporeal treatments compared to plasma from patients after extracorporeal granulocyte treatments (Mann-Whitney* U* test; *n* = 10; median/0.25–0.75 quartile).

**Figure 5:**
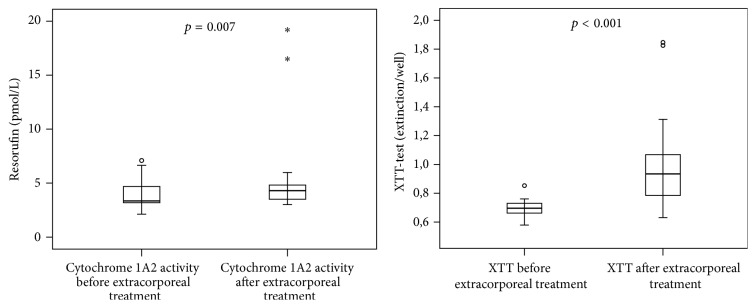
Activity of cytochrome 1A2 (metabolism of ethoxyresorufin to resorufin) and the XTT-test (dehydrogenases activity in the mitochondria) of HepG2/C3A cells (biosensor-test) incubated with plasma from septic patients before extracorporeal treatments compared to plasma from patients after extracorporeal granulocyte treatments (Mann-Whitney* U* test; *n* = 10; median/0.25–0.75 quartile). ^*∗*^Whisker.

In the cell based analysis of hepatotoxicity (biosensor), the HepG2/C3A cells were incubated with plasma of the patients before and after each extracorporeal granulocyte treatment. The cell-count and vitality (Figure 3), the synthesis of microalbumin (Figure 4), and the activities of cytochrome 1A2 and mitochondrial dehydrogenases (XTT-test, Figure 5) increased significantly during extracorporeal treatment. In addition, the values of lactatedehydrogenase (LDH) were significantly lower after extracorporeal treatment than the values of LDH before extracorporeal treatment (Figure 4). Only in survivors were significant increases of all biosensor parameters observed between day 1 (before extracorporeal treatment) and day 3 after extracorporeal granulocyte treatment (*p* < 0.05, data not shown), with the exception of LDH (significant decrease in survivors).

## 4. Discussion

The therapy with the extracorporeal granulocyte bioreactor system led to a reduction of norepinephrine dosage; moreover, the liver cell function and the viability in the biosensor-test were improved. We showed as before in the first clinical study [25] a good compatibility of the system. No significant changes in coagulation markers or any hemorrhages and signs of hemolysis were observed.

The extracorporeal granulocyte therapy influenced the immune system of the treated patients. During the six-hour treatment, an increase in the number of leukocytes and a decrease of procalcitonin were observed in the sera of the patients. The expression per cell of HLA-DR on monocytes increased significantly from inclusion to day four; additionally, between day 1 (before extracorporeal treatment) and day 3 after extracorporeal treatment, the values of IL-6 and IL-8 increased slowly. Immunomodulation has been introduced as a supportive therapy to overcome immune system dysfunction and could show positive impact on survival of patients with severe sepsis in some studies [35] but failed in a number of other studies [36, 37]. Extracorporeal blood detoxification methods have also been suggested to successfully influence immune imbalances and subsequently clinical course and outcome of multiorgan failure and sepsis [8, 9, 38, 39], because immunosuppression seems to be the main course of mortality of patients with sepsis and multiple organ failure [40].

The transfusion of granulocyte preparations (GTx) failed to improve survival in sepsis and neutropenic patients [41, 42]. However, there is some indication that steroid- or G-CSF-stimulated high-yield granulocyte donations might result in better survival in severe infections associated with neutropenia and cancer [42, 43]. In order to deploy the beneficial features of neutrophils such as phagocytosis of cellular debris, antigenic material, or pathogens and at the same time to circumvent the possible damaging local effects of systemically transfused neutrophils, a bed-side bioreactor was built, which uses granulocytes in a strictly extracorporeal mode. The bioreactor-cells are retained in the extracorporeal system and discarded after the treatment.

The 28-day mortality was 40% in the studied patients, consisting of nine patients with septic shock and one patient with severe sepsis, and is comparable with the results of other studies [1, 2]. Patients 2 and 4 died after reduction of therapy on palliative care (on the very same day and during the observation time). During the first extracorporeal treatment, patient 6 died; an autopsy revealed an advanced and longer existing ischemia of the bowel. No conclusions about survival can be drawn based on this (uncontrolled) study. The aim of the study was to document the impact of the extracorporeal granulocyte treatment on the dosage of norepinephrine in patients and the liver function using extended hemodynamic monitoring, dynamic measurement of the liver function with the LiMON-System, and, additionally, cell based analysis of hepatotoxicity of patient's plasma. Both need of vasopressors and the occurrence of liver dysfunction or failure are well known to impair the prognosis of patients with severe sepsis [5–7, 44]. The general idea of this work was to postulate that a new therapy of severe sepsis, for example, extracorporeal treatments, should be proven for a positive influence of hemodynamics and the liver in the septic organism.

Under reduction of vasopressor administration, the mean arterial pressure was stable and the cardiac index and the stroke volume index increased slightly during treatments measured with a dynamic monitoring system (PiCCO, 27). These results are the second report of a decreasing dosage of norepinephrine in patients using this extracorporeal granulocyte treatment system [25].

In this study, different parameters of liver function and hepatotoxicity were measured. The static parameter bilirubin was significantly lower in survivors than in nonsurvivors at the beginning of the extracorporeal treatment. During the observation time of 28 days, however, no significant differences of bilirubin-values were seen between the survivors and nonsurvivors and during extracorporeal treatments. Bilirubin is known as a good prognostic parameter for liver dysfunction or failure. In contrast, for acute changes in liver function, bilirubin seems to be not a valuable parameter [45, 46].

For the measurement of acute impairment of the liver function, dynamic measurement with tracer-substances was introduced [28]. We used the LiMON-System based on the indocyanine green plasma disappearance rate (PDR) and calculated the ICG-PDR cardiac index ratio. In accordance with other studies [47, 48] we found significant lower values in nonsurvivors than in survivors on day 1 before extracorporeal treatment and an increase of PDR from day 1 to day 7 only in surviving patients. With regard to clinical characteristics, three patients of the nonsurvivor group developed a (sepsis-induced) liver failure in the course of the disease; therefore, the time between beginning of shock and beginning of extracorporeal treatment was 5.5 days. During the extracorporeal treatments, however, no significant increases of PDR-values or ICG-PDR cardiac index ratio were observed. The cause of this could be the low number of patients. Patients with markedly decreased PDR-values (<8%) did not respond well to the extracorporeal granulocyte treatment. The PDR of indocyanine green provides information for the assessment of mortality and morbidity in critically ill patients, in patients with severe sepsis, and in patients underlying cardiac surgery [47–50]. Limitations of the LiMON-System are the nonexistent discrimination between liver function and perfusion of the splanchnic tract and false data in cases of hyperdynamic sepsis and hyperbilirubinaemia [51, 52].

In the cell based analysis of hepatotoxicity (biosensor; 29, 30), the HepG2/C3A hepatocyte cell line was incubated with plasma of the patients before and after each extracorporeal granulocyte treatment. During the extracorporeal treatments, a significant increase of vitality and function of the test cells was seen. These results suggest a positive impact of the extracorporeal granulocyte treatment on the liver cell vitality and function measured in this indirect cytotoxicity test. The reasons for this are still not clear but may be a result of reduction of toxins and drugs and the influence of chemokines and cytokines of the patients by the use of the extracorporeal bioartificial therapy. These factors are known to impair the cell functions and viability in HepG2/C3A cells [53–56]. Proinflammatory cytokines, for instance, are known to cause a dysfunction of mitochondria [50], to downregulate albumin synthesis [54], and to diminish function of some P450 cytochromes like CYP 1A2, CYP 2E1, and CYP 7A1 [55, 56].

Using the cell based analysis of hepatotoxicity of patient's plasma, only in survivors were significant increases of all biosensor parameters observed between day 1 (before extracorporeal treatment) and day 3 after extracorporeal granulocyte treatment. These data suggest a prognostic property of the biosensor in patients with severe sepsis, similarly to the PDR of indocyanine green [47]. In a previous prospective clinical study, the hepatotoxicity of plasma from septic and nonseptic patients was tested with this biosensor [29]. We found that the plasma of patients with septic shock impaired cellular functions and viability of HepG2/C3A cells. These values of biosensor parameters were increased only in survivors compared to nonsurvivors in this study. Additionally, only in the septic shock group were negative correlations found between cytochrome 1A2 activity and synthesis of albumin with APACHE II and SOFA scores (correlation coefficient: <0.7, *p* < 0.05).

## 5. Conclusions

In summary, there are three significant findings based on the results of the present study: (a) extracorporeal granulocyte treatment led to immunomodulation and a reduction of norepinephrine-dosage in patients during extracorporeal therapy, (b) patients with markedly decreased indocyanine green PDR-values did not respond well to the extracorporeal granulocyte treatment and an increase of PDR was only seen in survivors, and (c) a positive impact on the viability and function of hepatocyte senor cells (hepatotoxicity test of patient's plasma) were seen during the extracorporeal treatments.

Further studies should focus on changes of the hemodynamic system and the liver function in septic patients because of the prognostic value of these organ systems. Additionally, studies investigating the role of extracorporeal cell-therapies for sepsis are encouraged and should also address the influence on the survival of patients with severe sepsis.

## Figures and Tables

**Table 1:** Patients characteristics, illness severity, premorbidity, and clinical outcome for the study cohort (*n* = 10).

Patient	Major diagnoses at inclusion	Source of infection	Organ failure	Premorbidity	Age (years)	Sex (m)	Hospital survival
1	SS, ALI	Pneumonia, bacteremia, infection of foot, and thoracic empyema	ARF, ALI	Diabetes mellitus, chronic heart failure	66	m	Died (day 62)
2	SS, ALI, and endocarditis	Bacteremia, infection of sternum	ARF, ALI, and liver failure	Diabetes mellitus	72	m	Died (day 2)
3	Severe sepsis, thoracic empyema	Pneumonia, bacteremia	ALI	Alcohol abuse	33	m	Survived
4	SS, colitis	Peritonitis, colitis	ARF, ALI, DIC, and liver failure	Chronic heart failure	78	m	Died (day 4)
5	SS, ALI	Pneumonia, urinary tract infection	ARF, ALI	IHD, immunosuppression	59	m	Survived
6	SS, spondylodiscitis	Pneumonia, bacteremia, spondylodiscitis, and infection of arm	Liver failure, ALI, and ARF	COPD	67	m	Died (day 1)
7	SS, urosepsis	Pneumonia, bacteremia, and urinary tract infection	ALI, ARF, and DIC	Diabetes mellitus, IHD	76	m	Survived
8	SS, peritonitis	Bacteremia, pneumonia, and peritonitis	ALI	COPD, alcohol abuse	62	m	Died (day 22)
9	SS, perforated aortic aneurysm	Bacteremia, pneumonia, and peritonitis	ARF, ALI	IHD, cancer	83	m	Survived
10	SS, after urgent ACB surgery	Pneumonia	ALI	Diabetes mellitus, COPD, and IHD	57	m	Survived

ACB: aortocoronary bypass.

ALI: acute lung injury.

ARF: acute renal failure.

COPD: chronic obstructive pulmonary disease.

DIC: disseminated intravascular coagulation.

IHD: ischemic heart disease.

m: male.

SS: septic shock.

**Table 2:** Age, scores, and results of laboratory parameters, of hemodynamic monitoring (PiCCO-System), and of dynamic measurement of the liver function (LiMON-System) at inclusion or on day 1 (before granulocyte therapy) of survivors and nonsurvivors (median/0.25–0.75 quartile).

	Survivors (*n* = 5)	Nonsurvivors (*n* = 5)	Statistical significance (*p*)
Age (years)	59 (57–76)	67 (66–72)	n.s.
Surgery/no surgery	4/1	5/0	
APACHE II at ICU arrival/at inclusion	20.5 (17.8–25)/22 (22–34)	27 (27–33)/30 (28–32)	n.s.
SOFA at inclusion	8 (6–10)	14 (13–14)	n.s.
SAPS II at inclusion	47 (44–62)	74 (73–78)	**0.032**
MODS at inclusion	8 (7–12)	12 (11–13)	n.s.
Bilirubin (*µ*mol/L)	12 (11–17)	44 (30–145)	**0.016**
ALAT (U/L)	32 (20–45)	37 (30–219)	n.s.
ASAT (U/L)	62 (44–74)	105 (52–669)	n.s.
Ammonia (mmol/L)	36 (32–62)	39 (38–61)	n.s.
PCT (ng/mL)	7.3 (0.5–43)	10.4 (7.4–14.6)	n.s.
Leukocytes (GpT/L)	18.1 (14.8–18.1)	16.2 (13.9–22.2)	n.s.
Thrombocytes (GpT/L)	159 (89–278)	125 (47–269)	n.s.
Prothrombin time as INR	1.09 (1.01–1.1)	1.32 (1.11–1.33)	n.s.
Activated partial thromboplastin time (s)	36 (33–49)	48 (45–54)	n.s.
Creatinine (*µ*mol/L)	143 (79–238)	178 (165–213)	n.s.
Urea (mmol/L)	15.7 (5.7–16)	14.7 (13.8–24.9)	n.s.
Lactate (mmol/L)	1.0 (0.9–1.8)	2.0 (1.6–2.1)	n.s.
Complement C3 (g/L)	1.1 (1.0–1.8)	0.7 (0.5–1.0)	n.s.
Complement C4 (g/L)	0.5 (0.4–0.9)	0.4 (0.2–0.5)	n.s.
HLA-DR/CD-14 positive cells (expression/cell)	6413 (5890–6970)	8980 (7640–9590)	n.s.
Cardiac index (L/m^2^/min)	3.5 (3.3–4.0)	2.5 (2.5–3.4)	n.s.
Stroke volume index (mL/m^2^)	48 (43–51)	29 (28–39)	n.s.
MAP (mmHg)	92 (84–93)	71 (69–85)	n.s.
Norepinephrine (*µ*g/kg/min)	0.13 (0.06–0.21)	0.36 (0.29–0.51)	**0.032**
ICG-PDR on day 1 (%)/ICG-PDR/CI	14.4 (13.3–17.2)/5.6 (4.9–5.7)	5.3 (2.5–11.9)/2.9 (1.1–2.9)	**0.048/0.05**

ALAT: alanine aminotransferase.

APACHE: Acute Physiology and Chronic Health Evaluation.

ASAT: aspartate aminotransferase.

CI: cardiac index.

HLA: human leukocyte antigen.

ICG-PDR: indocyanine green plasma disappearance rate.

MAP: mean arterial pressure.

MODS: multiorgan dysfunction syndrome (score).

n.s.: not (statistically) significant.

PCT: procalcitonin.

SAPS: simplified acute physiology score.

SOFA: sequential organ failure assessment (score).

**Table 3:** Cytokines values before the first extracorporeal granulocyte therapy, after the second extracorporeal granulocyte therapy, and after 7 days (median/0.25–0.75 quartile).

Cytokine	Before first extracorporeal therapy (day 1) (*n* = 10, pg/mL)	After second extracorporeal therapy (day 3) (*n* = 8, pg/mL)	After 7 days (day 8) (*n* = 7, pg/mL)
IL-6	57 (41–159)	78 (45–154)	28 (26–62)^#^
IL-8	40 (28–53)	57 (38–118)	30 (29–36)
TNF-alpha	30 (22–40)	22 (19–26)	15 (13–17)^*∗*#^
IL-10	15 (8–20)	11 (8–13)	3 (3–8)

^*∗*^Statistically significant (*p* = 0.028) compared to day 1 (before first extracorporeal treatment).

^#^Statistically significant (*p* < 0.05) compared to the end of second extracorporeal treatment.

IL: interleukin.

TNF: tumor necrosis factor.

**Table 4:** Hemodynamic parameters, central venous oxygen saturation, dynamic measurement of the liver function (LiMON-System), and lactate before and after the extracorporeal granulocyte therapy (median/0.25–0.75 quartile) measured with the PiCCO-System (under significant reduction of norepinephrine; see Figure 2).

	Before extracorporeal therapy (*n* = 9)	After extracorporeal therapy (*n* = 8)	Statistical significance
CI (L/m^2^/min)	3.1 (2.3–3.8)	3.5 (2.9–3.7)	n.s.
SVI (mL/m^2^)	42 (33–56)	45 (40–52)	n.s.
MAP (mmHg)	73 (65–83)	74 (66–79)	n.s.
Heart rate (beat/min)	73 (67–95)	76 (70–85)	n.s.
SVRI (dyne × sec × cm^−5^/m^2^)	1510 (1150–1730)	1290 (1130–1760)	n.s.
ITBVI (mL/m^2^)	1060 (930–1160)	1000 (960–1120)	n.s.
EVLWI (mL/kg)	7.6 (6.8–9.4)	7.5 (6.5–8.3)	n.s.
cvSpO2 (%)	73.5 (68.4–76.5)	71.5 (68.6–79.1)	n.s.
Lactate (mmol/L)	1.5 (1.2–3.0)	1.3 (1.0–2.7)	n.s.
ICG-PDR (%)/ICG-PDR/CI	13.8 (11.9–15.6.)/4.4 (3.1–5.1)	14.2 (12.4–17.1)/4.6 (3.1–4.8)	**n.s./n.s.**

CI: cardiac index.

cvSpO2: central venous oxygen saturation.

EVLWI: extravascular lung water index.

ICG-PDR: indocyanine green plasma disappearance rate.

ITBVI: intrathoracic blood volume index.

MAP: mean arterial pressure.

n.s.: not (statistically) significant.

SVI: stroke volume index.

SVRI: systemic vascular resistance index.

## Abbreviations

ACT: Activated clotting time
ALT: Alanine transaminase
AST: Aspartate transaminase
APACHE: Acute Physiology and Chronic Health Evaluation (Score)
G-CSF: Granulocyte-colony stimulating factor
GTx: Granulocyte transfusion
HLA-DR: Human leukocyte antigen DR
IL: Interleukin
INR: International Normalized Ratio
MAP: Mean arterial pressure
MODS: Multiple Organ Dysfunction Score
PCT: Procalcitonin
PDR: Plasma disappearance rate
SAPS: Simplified Acute Physiology Score
SOFA: Sequential Organ Failure Assessment (Score)
TNF: Tumor necrosis factor.
